# Epidemiology of Imported Malaria in the Mediterranean Region

**DOI:** 10.4084/MJHID.2012.031

**Published:** 2012-05-07

**Authors:** Silvia Odolini, Philippe Gautret, Philippe Parola

**Affiliations:** 1 Institute for Infectious and Tropical Diseases, University of Brescia, Brescia, Italy; 2 Service des Maladies Infectieuses et Tropicales, Hôpital Nord, APHM, Aix Marseille Université, Marseille, France

## Abstract

Malaria is one of the most widespread infectious diseases of our time, causing 655 000 deaths during 2010 (WHO), most of them in sub-Saharan Africa and under the age of 5. During the last few years an increasing number of imported malaria cases is reported in Europe and Mediterranean countries, probably supported by the increasing number of international travel in association with the important influx of immigrants from malaria-endemic countries. Moreover, the presence of *Anopheline* vectors in Mediterranean countries, the returned infected travellers as a source of parasite and climate changes may result in the reappearance of malaria in countries where it was previously eradicated, such as Greece in recent years. Several cases of autochthonous malaria have recently been reported to support the need of an ongoing surveillance for mosquito control and an increased vigilance by health professionals. The aim of this paper is to critically review all the available literature about imported malaria in Mediterranean areas and the potential consequences of this.

## Introduction

In 2010 an estimated 3.3 billion people were considered at risk of malaria, especially in sub-Saharan Africa where 81% of cases and 91% of deaths are estimated to have occurred, with children under five years of age and pregnant women being most severely affected.^1^

It represents a serious health hazard for travellers to areas of endemicity. International travel is growing rapidly worldwide and this growth is particularly fast in the world’s emerging regions and tropical and subtropical areas^2^ reflecting in a significant number of imported malaria cases in countries in which the disease is not endemic. It is estimated that every year 10–15 million international travellers from Europe visit malaria endemic areas and 12000–15000 cases of malaria are imported into European Union countries with an average fatality rate of 0.4–3%.^3^ Numerous outbreaks and case reports serve as reminders that infections can be imported and/or transmitted in Europe by visiting or returning travellers. The purpose of this article is to review current knowledge on imported malaria in the Mediterranean Region (considering specific countries of European and Eastern Mediterranean Region according to WHO definitions) and the reappearance of *P.vivax* malaria in countries where it was previously eradicated.

## Imported Malaria Cases

Imported malaria has been described as “an infection that was acquired in an endemic area by an individual (either a tourist or indigenous native) but diagnosed in a non-endemic country after development of the clinical disease”.^4^ The increasing number of international travel in association with the significant influx of immigrants from malaria-endemic countries had a significant impact on malaria cases in developed countries.^5^

**Figure 1.**
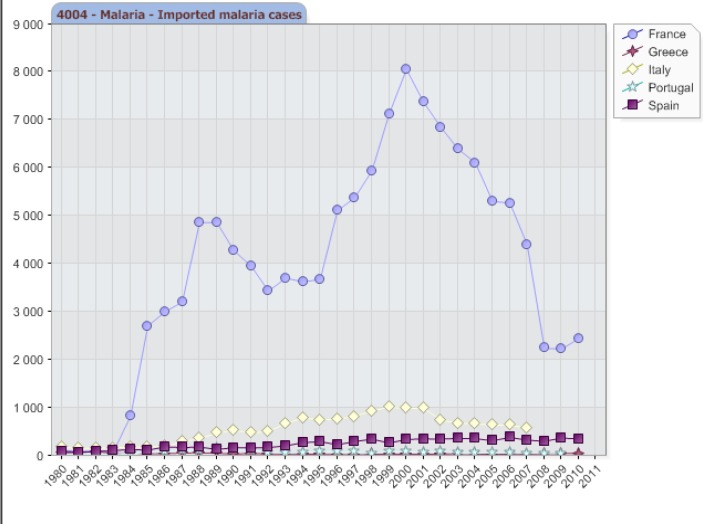
Imported Malaria cases in some European countries. WHO/Europe 2011, Centralized information system for infectious diseases (CISID)^31^. Available at http://data.euro.who.int/cisid/?TabID=281080. Accessed on January 25^th^, 2011.

Between 2001 and 2010, 45 countries in the European Region reported a decline in imported malaria cases and deaths, for reasons that have not yet been investigated, possibly reflecting malaria control activities in endemic countries,^1^–^3^ and a possible under-reporting of cases (**Figure 1**), but malaria in the Mediterranean regions remains an important travel medicine issue, given the large number of imported cases.

In the last annual epidemiological report of the European Centre for Disease Control and Prevention (ECDC), in fact, 6049 confirmed cases of malaria were reported in 2009 by 26 EU (European Union countries) and one EEA(European Economic Area)/EFTA(European Free Trade Association) countries in continental Europe, showing an increasing trend in the total number of malaria cases compared to 2008 (5912 confirmed cases). The main part (about 80%) of these cases were reported by France, United Kingdom, Italy and Germany. The prevalence of confirmed malaria cases was higher among males and in people aged 25–44 years and this likely reflects population travel patterns rather than other risk factors. The number of imported malaria cases in Europe is higher during the summer holiday months (June–October) and peaking in September with a lower increase in January, possibly related to the winter holiday period.^6^

In a study on ill returned travellers who presented in 2008 at EuroTravNet centers with a presumed travel associated condition, malaria was the most frequent diagnosis among febrile systemic illnesses, with a total of 371 cases of uncomplicated malaria reported and 12 cases of complicated malaria. The majority of *P.falciparum* cases were acquired in sub-Saharan Africa (SSA) by travellers who where visiting friends and relatives (VFRs) while the majority of *P.vivax* cases were acquired in South Central Asia and South America.^7^

A recent EuroTravNet study focused on travel related diseases in 2009, showed a slight increase, even if not statistically significant, in the total number of imported malaria cases in 2009 compared to 2008. *Plasmodium falciparum* was overall the most frequent diagnosis, but a particular increase in *Plasmodium vivax* cases in military patients coming from French Guyana seen at the Marseille clinic was observed. Also the number of pediatric malaria cases rose from nine in 2008 to 28 in 2009 and this was mainly due to children born in Europe who were travelling in malaria endemic countries (mainly in west Africa and Malawi) for visiting friends and relatives.^8^

Travellers visiting friends and relatives constitute the most significant group for malaria importation in developed countries that, due to their behavioral patterns and for behavioral and geographical reasons, are at high risk for infection. It is known that malaria partial immunity in VFRs who are resident outside malarious areas wanes with time resulting, especially after 12 years, in a more serious malaria clinical presentation.^9^ Several studies demonstrate also a lower use of malaria chemoprophylaxis in VFRs, probably also for socio-economic factors, in order to confirm the higher risk of disease in this particular group of travellers.^10^–^11^–^12^

It is also demonstrates a considerable variation in recommendations for malaria chemoprophylaxis among professionals in Europe, due to insufficient details about the type of travel and to the heterogeneity of national guidelines across Europe.^13^

It is reported that also among European travellers the use of chemoprophylaxis is still low. A sentinel surveillance of data collected during the period 1999–2000 among the member sites of the TropNet Europ shows that only 60.4% of European travellers compared to 77.2% of the immigrants travellers used malaria chemoprophylaxis and that only a minority of patients took drugs or drug-combinations appropriate for the drug resistance situation of malaria parasite at the respective destination.

The same study describes how many malaria cases originate from West Africa and affect travellers of African ethnicity who were principally travelling to visit friends and relatives. Several factors appear to determine the incidence of imported cases of malaria, including areas of endemicity visited, intensity of exposure, and success of prophylactic measures.^14^

Another study about all malaria cases seen in Italy from 2000 to 2006 identified some risk factors for acquiring malaria among Italian travellers: i) the lack of awareness of the risk: 119 cases of malaria were reported among Italian tourists in Kenya and other 21 tourists in the islands of Zanzibar who were staying in comfortable hotels, holiday resort and touristic villages, meaning that probably the only real risk is perceived for stays in rural areas, or they probably did not consider the risk at all ii) the period of the journey and seasonality of malaria transmission in a given country: the majority of malaria cases among Italian travellers occurred in winter while among VFR the majority of cases were reported from august to September; iii) reason of travel: most of malaria cases were reported among tourists (46%) and workers (54%); iv) chemoprophylaxis and drug resistance: malaria was reported in 27% of travellers who had taken chemoprophylaxis, but about 1/3 of these interrupted or suspended the chemoprophylaxis during the journey, and only 60% of the people who took adequate chemoprophylaxis resulted to have taken the correct drug for the country visited. In this study 5219 cases of malaria were reported, 5 were autochthonous and 5214 imported. Between 2000 and 2006 imported malaria cases fell from 977 to 630 respectively, with a total reduction of about 36%.^3^

Garcia-Villarrubia et al. in a recent population-based observational study on imported malaria in children less than 20 years of age resident in Barcelona (Spain), 174 cases of malaria were reported from January 1, 1990 to December 31, 2008. The majority of cases had travelled to Africa (83.9%) and of the 125 cases who were prescribed chemoprophylaxis, almost all did not take it correctly (96.8%). The most common reason for travel was VFR and *P.falciparum* was the species most commonly detected. They observed a significant increase in imported malaria incidence from 1990 to 2000 (p<0.001) and a decrease from 2001 to 2008 (p=0.01) but the analysis of the entire study period did not show a statistically significant decline (p=0.41). Factors associated with imported malaria among immigrant resident were travelling for VFR (OR:6.2 CI 1.9–20.2) and age 15–19 (OR:3.7 CI 1–13.3).^15^

In **table 1** are reported several studies on imported malaria in different Mediterranean regions.

Information about malaria risk and preventive measures should improve both among travellers and physicians, in order to reduce the probability of acquiring of malaria.

## Autochthonous Malaria Cases

Malaria cases are defined as autochthonous when local Anopheles transmits *Plasmodium* in countries in which malaria has already been eradicated.

It is estimated that 50 million new cases of malaria will occur by 2100, due to climate changes.^16^ Malaria was endemic in Europe until the mid 20^th^ century, since than it has been considered eradicated. Cases of indigenous transmission malaria have been reported over the last ten years, but sustained local transmission has not been identified to date. However in recent years the disease re-emerged in residual foci of Eastern Europe and, further more, the present climate change situation could increase mosquito vectorial capacity, especially in southern countries of Europe and the Mediterranean.^17^ It is known that the incidence of mosquito-borne diseases, including malaria, is among those diseases more sensitive to climate.^18^ Southern Europe is among the most risky regions for malaria resurgence, especially for *P.vivax* malaria resurgence, due to i) climate characteristics; ii) the proximity to Africa, which promotes migration from malaria endemic areas mainly due to job opportunities in European countries; iii) the wide presence of Anopheline vectors.^16^

**Figure 2.**
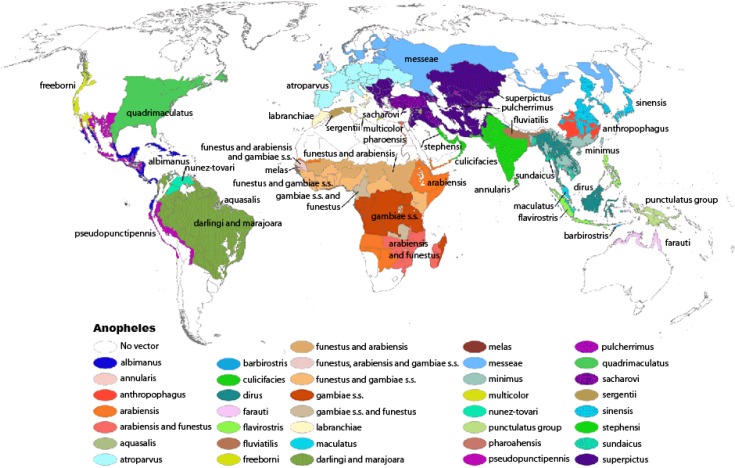
Global Distribution (Robinson Projection) of Dominant or Potentially Important Malaria Vectors. From Kiszewksi et al., 2004. American Journal of Tropical Medicine and Hygiene 70(5):486–498. Available at: http://www.cdc.gov/malaria/about/biology/mosquitoes/map.html

Anopheles atroparvus is an efficient malaria vector and it is widely distributed in Europe, except for some Mediterranean regions such as southern Italy, Greece and Turkey where Anopheles labranchiae and superpictus are prevalent (**Figure 2**). Several studies on the receptivity of the European vector Anopheles atroparvus reveal that it is not susceptible to the afro-tropical *P.falciparum* strains but is probably fully susceptible to infection by *P.vivax* strains imported from Africa.^16^

This makes allowance for a potential reappearance of malaria in countries where it was previously eradicated and the persistence of high numbers of imported malaria in the Mediterranean region becomes a phenomenon that requires attention.

The same is true also for non-malarious area in Saudi Arabia. It is known in fact that the risk of acquisition of malaria in Saudi Arabia is limited to the Southwestern part of the country, with the highest number of cases reported from Gizan and Asir regions. The Eastern Province of Saudi Arabia is considered free of malaria since 1978. Al-Tawfiq, in a retrospective study of the epidemiology of malaria in the Eastern Province of Saudi Arabia, reported 56 cases of imported malaria over the study period from January 1994 to June 2005. Most of the cases (78.7%) were acquired outside Saudi Arabia and the most frequent species diagnosed were *P.vivax* (54.4%), *P.falciparum* (43%) and P.malariae (1.8%). The presence of malaria-carrying vectors, especially Anopheles stephensi and Anopheles fluviatilis, has been reported putting on a state of alert the health authorities. Continuous monitoring of cases of malaria as well as an effective treatment is needed to prevent the reintroduction of malaria into this region.^19^

Another retrospective study on imported malaria in northern central Marocco highlights the potential risk or reappearance of malaria in this area. Since 2004 Marocco is considered malaria-free. In this study, El Ouali Lalami et al., reported 56 cases of imported malaria in the period 1997–2007, to underline the need to maintain high level of malaria monitoring and control in this area. The most frequent specie detected was *P.falciparum* (89%) followed by *P.vivax* (7%) and P.ovale (4%).^20^

**Figure 3.**
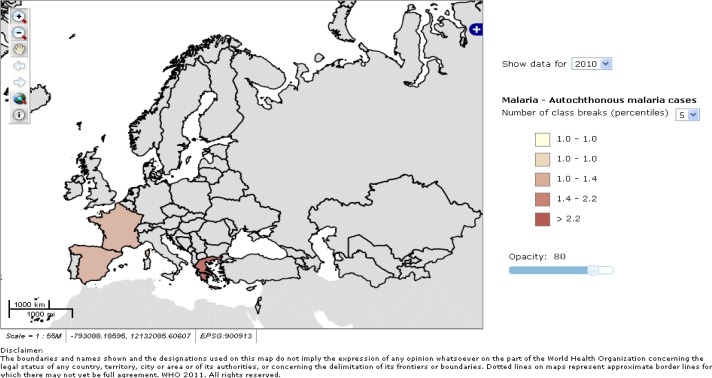
Autochthonous malaria cases in some European countries during 2010. WHO/Europe 2011, Centralized information system for infectious diseases (CISID)^32^. Available at http://data.euro.who.int/cisid/?TabID=281082. Accessed on January 25^th^, 2011.

In the last years several cases of autochthonous malaria have been reported in some Mediterranean countries (**Figure 3**), and these occur when an infected traveller infects local mosquitoes that can promote malaria transmission in people without history of travels in malaria endemic areas.

In summer 1997, one autochthonous *P.vivax* case occurred in Italy. It was diagnosed in Maremma (Tuscany) in a rural zone, which was known for the presence of Anopheles labranchiae. An investigation among people living close to the patient’s house was performed and a malaria case imported from India 3 months before and still carrying gametocytes was detected.^21^–^22^

The same happened in Corsica in August 2006, where a case of indigenous *Plasmodium* vivax malaria was diagnosed in the village of Porto (Department of South Corsica). It was the first case of autochthonous malaria transmission in France since 1972, and an imported *P.vivax* case in a traveller coming from Madagascar occurred the month before, suggesting a transmission by local Anopheles. The presence of Anopheles petragnani, Anopheles Claviger and Anopheles labranchiae in Corsica was detected.^23^

In 2010 the first indigenous cases of *P.vivax* malaria were reported in Aragon, Spain, where the vector Anopheles atroparvus is present,^24^ and in August 2011 a *Plasmodium* vivax malaria infection was diagnosed in a Romanian traveller returning from Greece. He had no history of travel to any malaria-endemic areas, he never travelled by plane and he didn’t live in the proximity of international airports. He worked intermittently in Greece for about six years (in Argos and Lakonia regions) and from November 2010 to July 2011 he always worked in Skala and Elos regions in agriculture. He only had a two-month trip to Italy (Sicily) from September to October 2010. It is known that in Greece several Anopheline vectors species are present, including A.sacharovi and A.superpictus, that are competent for *P.vivax*, facilitating the insurgence of autochthonous cases in this area.^25^

After this case, an increasing number of *P.vivax* cases in patients residing in Evrotas area were reported from Greek Health Authorities and, up to 27 September 2011, twenty cases of *P.vivax* infection in citizens without history of travel to malaria endemic areas were diagnosed. The majority of these cases were reported from Lakonia, a limited agricultural area of Evrotas, an area with a large population of non-documented migrant farm workers from malaria endemic-countries (i.e Pakistan and India). In the same area 16 more cases of *P.vivax* malaria were reported in migrants with unclear malaria importation status.

Greece was officially considered malaria-free in 1974, but sporadic cases without travel history were reported in 1991, 1999 and 2000.^26^ This highlights the role of travellers as sentinels for emerging infectious diseases and it has important implication for Public Health in order to recommend the adoption of measures to prevent mosquito bites for travellers to these areas and to enhance control measures among healthcare professionals and the general population.

## “Baggage Malaria”

The dramatic increase in international air transport facilitates the introduction of infected tropical vector insects in European countries through aircraft arriving at international airports, thus increasing the likelihood that vector-borne diseases may spread in this manner. The introduction of infected Anopheles mosquitos may support the transmission of malaria infection in people without a history of recent travel, promoting what is called “airport malaria” or “baggage malaria”,^27^ a particular form of autochthonous malaria. Since 1969, 30 cases of airport malaria have been reported in France, 2 during the summer of 2008.^28^ This phenomenon has important clinical implications due to the transmission of the infection in non-immune individuals and an often-important diagnosis delay because of lack of epidemiological information. Another case of *P.falciparum* malaria was diagnosed in a woman only after 6 days of intensive treatment for a sepsis syndrome and severe complications developed. The patient lived near Frankfurt airport and she did not have a history of foreign travel^29^. Imported malaria should always be thought in cases of fever of unknown origin without history of foreign travel.^30^

## Conclusion and Current Needs

The consistent numbers of imported malaria cases, which are a possible reservoir of infection, together with the occurrence of vector competent Anopheles species and the increasingly favorable climatic conditions, may induce the onset of autochthonous malaria cases in countries where malaria was previously eradicated, particularly in Mediterranean areas.

The development of a response plan for malaria covers all aspects, from surveillance, clinical management, laboratory diagnosis, entomological surveillance, vector control and communication in order to prevent transmission and control the disease in the long term.

There is the need to improve surveillance of malaria in order to identify possible indigenous transmission in European countries and to support assessment of prophylaxis recommendations for travel medicine.

Physicians should be aware about malaria risk and clinical features of malaria infection and should get used to suspect malaria also in patients without history of travel in malaria endemic areas, especially in the Mediterranean areas or in patients living near international airports.

In order to improve knowledge of travellers about malaria risk, services for immigrants and travellers should be more readily available; information about distribution and seasonality of malaria before the journey need to be discussed, compliance with adequate chemoprophylaxis and adoption of personal preventive measures should be enhanced and vector control measures around the perimeter of airport should be implemented.

Continuous monitoring to update information on epidemiological changes in areas of endemicity is essential.

## Figures and Tables

**Table 1.** Studies on imported malaria in some Mediterranean regions

**Author**	**Contry**	**Period of the study**	**Number of imported malaria cases reported**	**Prevalence of Plasmodium species reported**
**Garcia-Villarrubia M et al., 2011^15^**	Barcelona (Spain)	1990–2008	174	*P.falciparum* (69,5%), *P.vivax* (14.4%), *P.ovale* (5.7%), *P.malariae* (4.6%), Mixed (2.9%), *Plasmodium spp* (2.9%)
**Espinosa-Vega E et al., 2011^33^**	Gran Canaria Island (Spain)	1993–2006	184	*P.falciparum* (84.1%), P.vivax (11.8%), P.ovale (2.4%), P.malariae (1.2%), Mixed (0.6%)
**Rey S et al., 2010^34^**	Southern Madrid (Spain)	2005–2008	57	*P.falciparum* (94.7%)*, P.ovale* (5.3%)
**Romi R et al., 2010^3^**	Italy	2000–2006	5219 (5214 imported, 1 autochthonous)	*Plasmodium falciparum* (83%), *P.vivax* (8%), *P.ovale* (7%), *P.malariae* (2%), Mixed (0.3%)
**Al-Tawfiq JA, 2005^19^**	Eastern Province of Saudi Arabia	1994–2005	56	*P.vivax* (54.4%), *P.falciparum* (43%), *P.malariae* (1.8%)
**El Ouali Lalami A et al., 2009^20^**	Northern Central Morocco	1997–2007	56	*P.falciparum* (89%), *P.vivax* (7%), *P.ovale* (4%)
**Castro L et al., 2004^35^**	North of Portugal	1993–2002	140	*P.falciparum* (60.4%), *Plasmodium spp.* (22.9%) *P.vivax* (14.6%), *P.ovale* (2.1%)
**Pistone T et al., 2010^36^**	Bordeaux (France)	2000–2007	526	Prevalence of *P.falciparum*
**Parola P et al., 2005^37^**	Marseille (France)	2001–2003	352 (240 adults and 112 children)	*P.falciparum* (88.3% adults, 75.9% children), *P.vivax* (4.2% adults, 4.5% children)*, P.ovale* (2.5% adults, 7.1% children), *P.malariae* (0.0% adults, 0.9% children), *Mixed* (4.2% adults, 9.8% children)
**Parola P et al., 2007^38^**	Marseille (France)	2004–2006	248	*P.falciparum* imported from Comoros
**Aoun K et al., 2010^39^**	Tunis (Tunisia)	1999–2006	98	*P.falciparum* (71.4%), *P.ovale* (19.4%)
**Anis E et al., 2004^40^**	Israel	Annually	60–100	*n.a*
**Hammadi D et al., 2009^41^**	Algeria (south of the country)	1980–2007	300 (all autochthonous cases)	*n.a*
**Alvero O et al., 2009^42^**	Bursa (Turkey)	2006–2008	9	*P.vivax (*66.7%*), P.falciparum (*33.3%)
**Peric D et al., 2009^43^**	Croatia	1987–2006	201	*P.falciparum* (64.7%), *P.vivax* (19.9%), *P.malariae* (2.0%), *P.ovale* (0.5%), Mixed (6.0%)
